# Influence of clay mineral content on mechanical properties and microfabric of tailings

**DOI:** 10.1038/s41598-022-15063-3

**Published:** 2022-06-23

**Authors:** Chao Zhang, Zhenkai Pan, Hongwu Yin, Changkun Ma, Lei Ma, Xueting Li

**Affiliations:** 1 State Key Laboratory of Geomechanics and Geotechnical Engineering, Institute of Rock and Soil Mechanics, Chinese Academy of Sciences, Wuhan, 430071 Hubei China; 2 University of Chinese Academy of Sciences, Beijing, 100049 China; 3 Guangzhou Expressway Co., Ltd, Guangzhou, 510555 Guangdong China

**Keywords:** Civil engineering, Geology

## Abstract

Clay mineral content has an important influence on the mechanical behaviour of tailings, and this mechanical behaviour significantly affects the stability of tailings dams. X-ray fluorescence (XRF) and X-ray diffraction (XRD) tests were carried out on tailings from three different regions. The chemical and mineral composition of the tailings are analyzed. The strength and failure deformation of the tailings were studied by laboratory triaxial compression tests. The effect of clay content on the behaviour of tailings was investigated. The microfabric of tailings sample was examined with the scanning electron microscope (SEM) and nitrogen adsorption tests. The results show that the confining pressure corresponding to the samples exhibiting strain hardening increases with the increase of the content of clay minerals. The cohesion of tailings increases linearly, and the specific surface area decreases as the content of clay minerals increases. Nitrogen adsorption test results reveal from a microscopic point of view that changes in pore structure are associated with the content of clay minerals. The higher the content of clay minerals is, the higher the proportion of micropores is (aggregated interior). Macroscopically, the overall porosity decreases and the size of the pores increases with clay content, which will directly affect the mechanical properties of tailings.

## Introduction

Tailings are the waste materials discharged from mineral beneficiation plants. They are generated by grinding and beneficiation of the ores under specific economic and technical conditions. When dealing with tailings, the tailings pond is a necessary facility to maintain mine production. However, it is also a major source of hazard for the mine^1^. Mining companies in more than 100 countries exploit minerals and metals. To meet the needs of economic and social development, the number of tailings dams is increasing. When a tailings pond fails, it may cause serious losses to lives and property, and may pose a severe threat to the environment^2^. For example, on the night of September 8, 2000, a dam failure occurred at Boliden´s Aitik mine, close to Gallivare, Sweden. Contaminated water was released, polluting nearby rivers^3^. On April 30th, 2006, a failure occurred in a gold mine tailings impoundment in Shanxi province, northwest China, causing 17 deaths, 2 missing and 5 injuries, 76 houses destroyed and flooded^4^. In 2009, the gold tailings dam failure at Karamken, the Russian Far East, caused the release of the amassed water-saturated tailings. The outburst of the accumulated deposits spilt large quantities of toxic elements. They poisoned groundwaters and caused severe damage to the local riverine ecosystem and fishery, as well as destroying the nearby town, with human casualties^5^. On January 25th 2019, a tailings pond burst at Brumadinho City, Brazil. About 11.7 million m^3^ of a tailings-mud mixture was released, destroying 300 km of the Paraopeba River toward the São Francisco River^6,7^. As a result of multiple very serious accidents, the safety of tailings dams has received increasing attention from governments and communities with mining operations.

Although the influence of mineral composition on mechanical properties is not given the primary consideration in the literature, it is an important factor affecting mechanical properties^8^. It is related to the stability of tailings dams. At present, the influence of mineral composition on mechanical properties is a research topic for a wide range of geotechnical materials. Song and Hong^9^ investigated the effect of clay minerals on the suction of unsaturated soils by using an automated soil–water characteristic curve apparatus. They found that unsaturated characteristics considerably depend on the clay mineral composition and particle gradation. Ni and Huang^10^ used an oscillating shear rheometer and zeta analyzer to explore the effect of mineral composition on the viscoelastic properties of soil. Their results showed the sensitivity of the viscoelastic properties to montmorillonite was more important than that of kaolin or illite. Hu et al.^11^ studied the effect of mineral composition on mechanical properties of the Cox argillite by micro-indentation and mini-compression tests. They found that elastic modulus and shear strength decrease with the increase of clay content. Vazquez et al.^12^ analyzed the mechanical strength of granites with different lithologic facies, that is, varying mineral components. They also discovered that there is a significant statistical correlation between some quantitative lithofacies variables and mechanical strength characteristics of the granites.

Tailings are the parts with the lowest content of valuable target minerals after beneficiation. Besides the basic soil compositions, they also include heavy metal oxides and soluble acid substances, indicating that their composition is complex. Much research has been conducted to study the mechanical properties of tailings, for instance, mechanical behaviour and particle crushing characteristics of copper tailings under high confining pressure^13^, thermal conductivity and unconfined compressive strength of a fly ash-stabilized gold tailings^14^, and the uniaxial compressive strength and deformation modulus of iron tailings under freezing^15^. However, only few studies have been performed on the mechanical properties of tailings with different mineral compositions. Furthermore, many stacked tailings contain precious and rare metals that have not been recovered, which can be a valuable resource for secondary exploitation and utilization. Therefore, it is very important to study the influence of mineral composition on the mechanical properties of tailings. Since it can ensure the stability of tailings dams and their subsequent secondary utilization.

The motivation of this paper is mainly to study the effect of clay content on mechanical properties and microfabric of tailings. We carried out Consolidated Undrained tests on three different tailings. The strength and deformation of tailings with different clay content are described. The results provide a theoretical reference for the stability study of tailings dams with different attributes. We plan to study the effect of mineral types on mechanical behaviour and microfabric of the tailing samples in future research.

## Test materials and methods

### Tailings material properties

**Figure 1:**
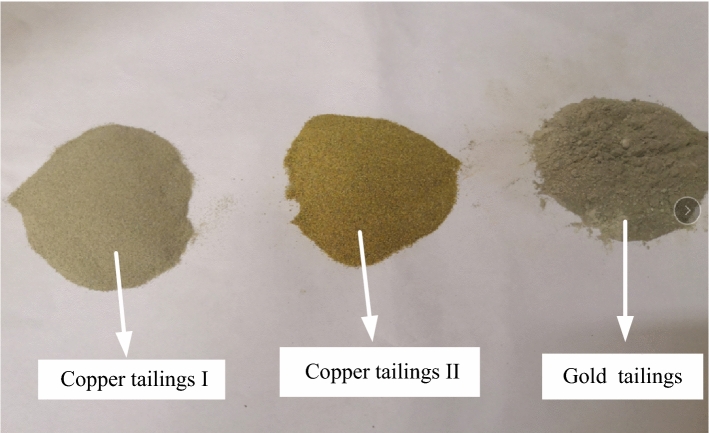
Original tailings samples.

**Figure 2:**
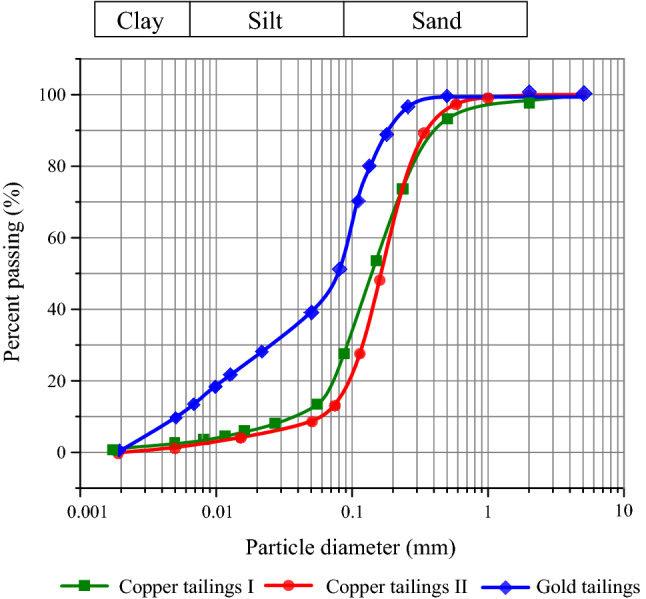
Particle size distributions of original tailings.

The tailings materials used in this study were collected from the dry beach surface of Dexing and Fengshuling copper tailings dams in Jiangxi Province, and Longnan gold tailings dam in Gansu Province, China. The three types of tailings are named copper tailings I (CTI), copper tailings II (CTII) and gold tailings (GT), respectively, to simplify the narrative. The original tailings are shown in Fig. 1. Based on classification and identification of soils^16^, the original CTI is light grey, with loose particles, and these tailings have fluid consistency after adding water. The colour of CTII is golden yellow and they have a more pellet feeling. They show a plastic state when in a small amount of water, indicating that their minerals have weak hydrophilic capacity. The GT is charcoal grey with a distinct particle aggregation in water and a high plastic limit. Moreover, since the gold has a higher value, more cost has been spent on grinding during the beneficiation, resulting in finer tailings. The plasticity indexes of tailings are in descending order of GT, CTI and CTII. The particle size distribution curves of the original tailings are shown in Fig. 2. CTII has the highest sand-sized particles content, and the particle size distribution curves of CTI and CTII are relatively close to each other. The GT contains more silt-sized and clay-sized particles. The physical and mechanical parameters of the tailings are summarized in Table 1, indicating that CTI and CTII have a poor gradation and GT is a well-graded material according to the Unified Soil Classification System^17^. The GT has a relatively smaller dry density with higher Atterberg limits because of its high content of fine particles and high natural water content.

**Table 1:** Basic physical and mechanical properties of the tailings.

Sample ID	Mean particle size *d*_50_(mm)	Uniformity coefficient *C*_*u*_	Curvature coefficient *C*_*c*_	Moisture content (%)	Dry density (g/cm3)	Liquid limit *w*_L_ (%)	Plasticity limit *w*_P_ (%)
Copper tailings I	0.139	4.6	1.35	11.65	1.72	35.67	18.76
Copper tailings II	0.162	3.9	1.9	9.34	1.78	29.25	14.37
Gold tailings	0.065	11.7	1.23	15.9	1.65	39.23	20.71

### Sample preparation

**Figure 3:**
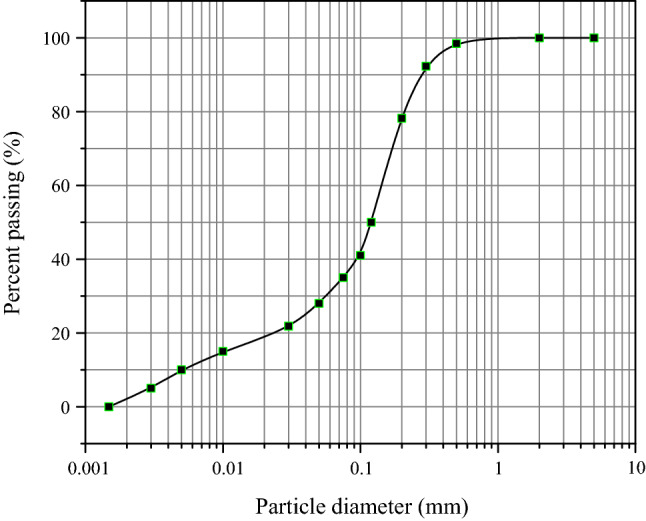
The particle size distribution curve of tailings samples tested.

Considering the effect of clay content on the mechanical properties of tailings, all tailings samples are set to the same particle size distribution to reduce the impact of any difference in size gradation on their mechanical properties. If the particle size distribution of the prepared sample were close to that of the original copper or gold tailings, it would have a significant difference from that of other tailings. Therefore, the average size gradation of all tailings was used for the testing grading. For this purpose, all original tailings are put in an oven at 105 °C and are dried for 24 h. And then, a series of sieves are used in the sieving machine to vibrate the tailings for 10 min. The new samples are mixed with the same volume percentages of particles of various sizes to prepare the normalized samples. The particle size distribution curve of these prepared samples is shown in Fig. 3.

The moist tamping method^18^ was adopted for sample preparation. Based on the particle size and the standard for soil test method^19^, the tailings samples for the triaxial test were 39.1 mm in diameter and 80 mm in height. Their void ratio can be obtained by the conversion of dry density and specific gravity, and its equation is as follows:

1 $$e = \frac{{G_{s} \rho_{w} }}{{\rho_{d} }} - 1$$

where $e$ is the void ratio, $G_{s}$ is the specific gravity, $\rho_{w}$ is the density of water,$\rho_{d}$ is the initial dry density of the tailings.

The specific gravity of the three tailings was 2.76, 2.78 and 2.77, respectively, with little difference. The initial dry density of the samples was set to 1.7 g/cm^3^. The void ratios for CTI, CTII and GT samples are 0.624, 0.635 and 0.629. Therefore, the initial void ratio of all tailings samples was the same. The liquidity indexes of CTI, CTII and GT samples are 0.23, 0.57 and 0.11 after saturation, respectively. According to the code for design and construction for international building (Day. 2009), the samples belong to the hard plastic, plastic, and hard plastic state respectively at sample saturation.

**Figure 4:**
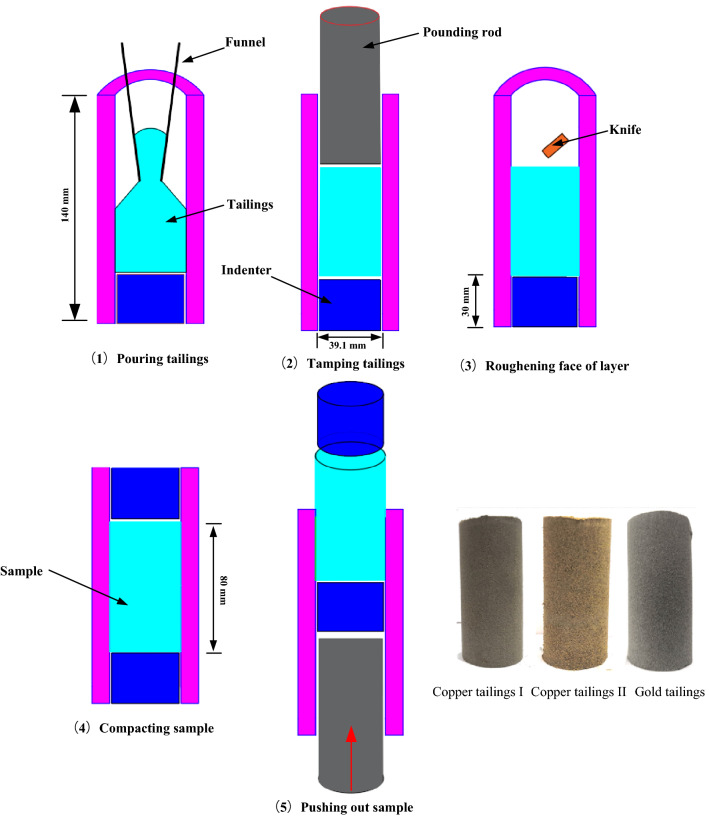
Sample preparation and prepared samples.

To mix the tailings materials uniformly, their water content for sample preparation was 15%. The mixture was sealed for 12 h after it was mixed. A lower indenter with 39 mm diameter and 30 mm height was placed at the bottom of the sampler. These prepared tailings were poured into the sampler in three identical layers to ensure sample uniformity. The tailings poured each time were tamped tightly to the same height. The interface between poured tailings layers was roughened with a knife. After preparing the sample in the sampler, another identical indenter was placed on the top of the sample. The sample preparation process and the prepared samples are shown in Fig. 4.

### Mineral compositions

X-ray fluorescence spectrometry (XRF) was used to analyze the chemical composition of the tailings. Their compositions are summarized in Table 2. The tailings mainly consist of oxides of silicon (Si), aluminium (Al), potassium (K) and iron (Fe), in the form of SiO_2_, Al_2_O_3_, K_2_O, and Fe_2_O_3_ in descending order by content, with the total amount reaching 90%, except for the gold tailings, with a slightly higher content of iron. The tailings contain a small number of heavy metal elements and sulfur oxide that easily forms acids. This will have a significant impact on the chemical and mechanical properties of the tailings.

**Table 2:** Chemical composition of tailings materials tested.

Major oxides (wt%)	Copper tailings I	Copper tailings II	Gold tailings
SiO_2_	63.141	69.344	59.723
Al_2_O_3_	20.056	18.226	19.061
K_2_O	4.869	4.009	4.438
Fe_2_O_3_	3.848	3.697	5.041
MgO	2.946	0.842	3.778
CaO	2.659	0.395	4.34
SO_3_	0.963	2.427	1.731
TiO_2_	0.921	0.722	0.89
CuO	0.245	0.02	–
MnO	0.086	0.066	0.163
BaO	0.051	0.017	0.071
Na_2_O	–	–	0.255
P_2_O_5_	0.013	–	0.206
Other heavy metal and rare metal oxides	0.215	0.235	0.466

**Figure 5:**
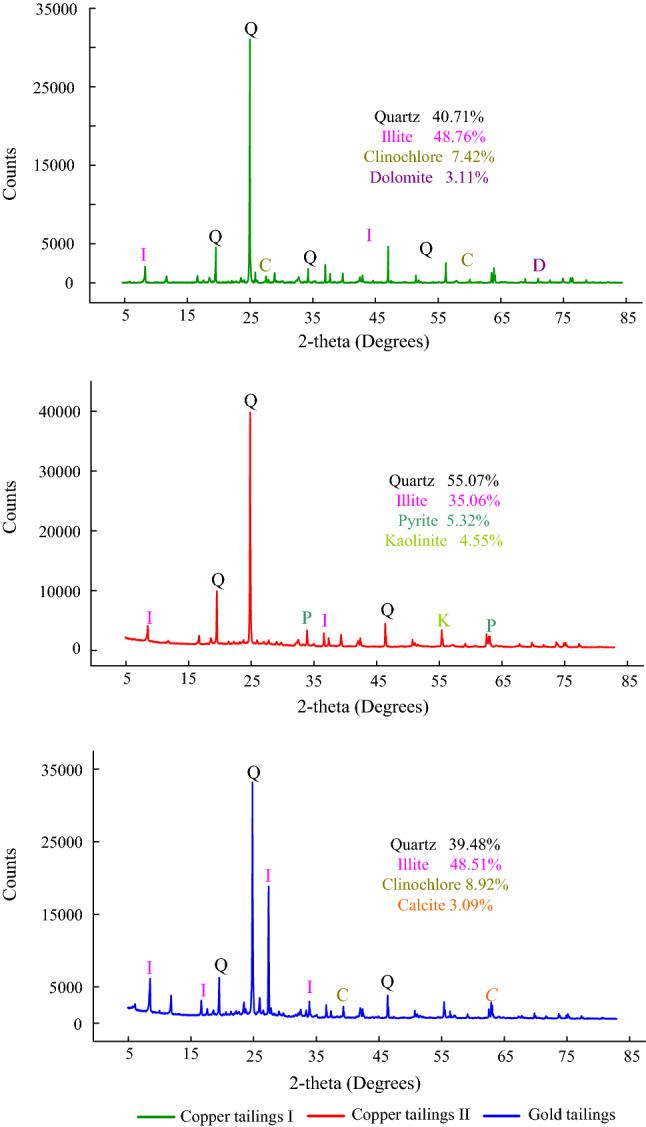
X-ray diffraction patterns of the three types of tailings [Q = quartz (00-046-1045); I = Illite (00-026-0911); C = Clinochlore (00-079-1270); D = Dolomite (00-074-1687); P = Pyrite (00-042-1340); K = Kaolinite (00-074-1784); *C* = Calcite (00-005-0586)].

An x-ray diffractometer (XRD) was used to investigate the mineral composition of the tailings. Considering that regular semi-quantitative XRD is less accurate, a quantitative Rietveld XRD method^20^ was used to analyse mineral contents. The XRD analysis results are shown in Fig. 5. The highest characteristic peaks of the three tailings are quartz.

### Triaxial compression test apparatus and test procedure

**Figure 6:**
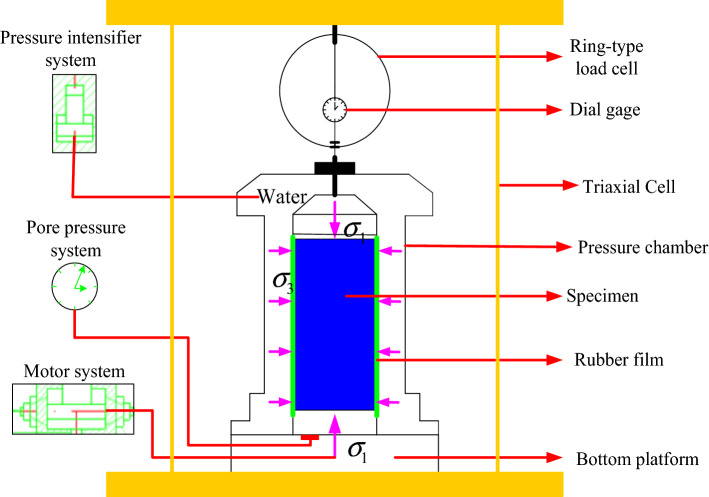
Schematic diagram of the triaxial test apparatus.

A strain-controlled triaxial test apparatus (SJ-1A.G) was adopted for the mechanical tests. Figure 6 shows a schematic diagram of the test apparatus. The main equipment of the apparatus consists of a confining pressure chamber, an axial load device, a speed control system, a confining pressure control system, and a test data acquisition system. It allows users to program and monitor axial load and sample volume changes in real-time, automatically collecting data. Three types of tests on soil materials are unconsolidated undrained shear (UU), consolidated undrained shear (CU), and consolidated drained shear (CD).

Triaxial tests on tailings samples were performed under confining pressures of 50, 100, 200, 300 and 400 kPa. According to the actual working conditions of the tailings dam, the samples were tested in an undrained shear test after isotropic consolidation. The samples were saturated by the vacuuming method. Because of the easy deformation of saturated tailings, the samples were perturbed and damaged when taken out of the saturator at room temperature (25 °C). To reduce disturbance and damage while installing the samples in the triaxial cell, the freezing method is used: the samples are frozen in preparation in a refrigerator. The completely frozen sample was installed in the triaxial cell as soon as possible after removing it from the refrigerator. The sample was put under backpressure to ensure sample saturation. The back pressure is set to 300 kPa. When pore pressure coefficient *B* reaches 0.95, the required confining pressure was applied to the sample for consolidation. The consolidation time is 4 h, and the shear speed is the axial strain of 0.06%/min according to the Standard test method for consolidated undrained triaxial compression test^21^. The test was ended when the axial strain reached 15%. When the sample was frozen, the expansion of the sample can be limited by lateral pressure, which this process does not change the porosity of samples. Therefore, freezing and thawing would not weaken the strength of the sample.

After the triaxial shear, the wholly dried tailings samples were used for scanning electron microscopy and energy dispersive x-ray spectrometry (SEM–EDS) with freeze-drying, which prevents fabric change induced by pore shrinkage as a result of air and oven drying. The sheared samples with a failure surface of 10 mm × 10 mm were placed instantly in liquid nitrogen for 2 min. The sample was sublimated in a vacuum drying cabinet for 24 h after freezing.

The nitrogen adsorption tests at low temperature on the sheared tailings samples were conducted using a Nova1000e apparatus produced by Quantachrome, USA. A sample of the small block was taken from the tailing samples after the triaxial test under the confining pressure of 100 kPa, and then it is freeze-dried for the nitrogen adsorption test. The nitrogen adsorption test uses pure nitrogen as the adsorbate. When liquid nitrogen is adsorbed, an adsorption film forms on the inner wall of a pore. The thickness of this film changes with the increase in relative pressure. When the adsorption pressure increases to a certain value, the cavity formed by the adsorption film will be filled with liquid nitrogen, then agglomeration occurs. Therefore, the adsorption pressure corresponds to the pore size, which can provide information on the pore size distribution of the tailings.

## Results and discussion

**Figure 7:**
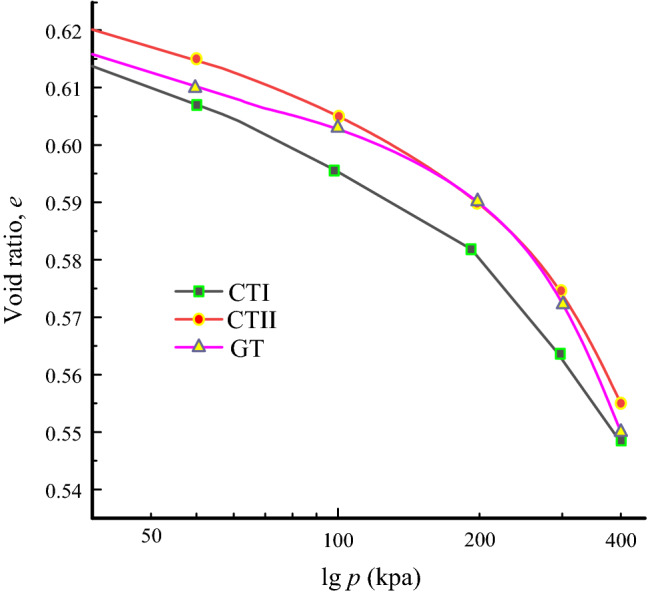
Compression curves of tailings samples with different mineral compositions.

Although the original samples were made with the same dry densities, the sample preparation and extraction may have changed their void ratio. This may result in different behaviour of samples. To verify the influence of the changed void ratios on the mechanical properties, the void ratios at the end of the triaxial tests are calculated. The *e*-lg *p* curves of the three groups of samples are shown in Fig. 7. The void ratios of the three tailings are similar after the triaxial tests. The differences in the void ratio are less than 1% under all confining pressures. Therefore, the effect of the differences between the samples' void ratio on mechanical properties can be ignored. The void ratios for CTI, CTII and GT samples are 0.613, 0.621 and 0.616, respectively, after sample preparation. It indicates that the freezing method for sample preparation leads to a decrease of the void ratio.

The main minerals, quartz and illite, account for 90% of the mineral content in all three materials, and the other mineral components, with small contents, are different. By mineral composition classification, the mineral composition of tailings can be divided into clay minerals, non-clay minerals, and metal minerals. The mineral contents of the tailings are listed in Table 3. The clay mineral content of CTII, CTI, and GT increases in this order. This is related to their mechanical properties.

**Table 3:** Mineral contents of the three types of tailings according to mineral category.

Tailings species	Mineral category			Total (wt%)
	Clay minerals	Non-clay minerals	Metallic minerals	
CTI	56.18	43.82	–	100
CTII	39.61	55.07	5.32	100
GT	57.43	42.57	–	100

### Influence of clay content on stress–strain characteristics

**Figure 8:**
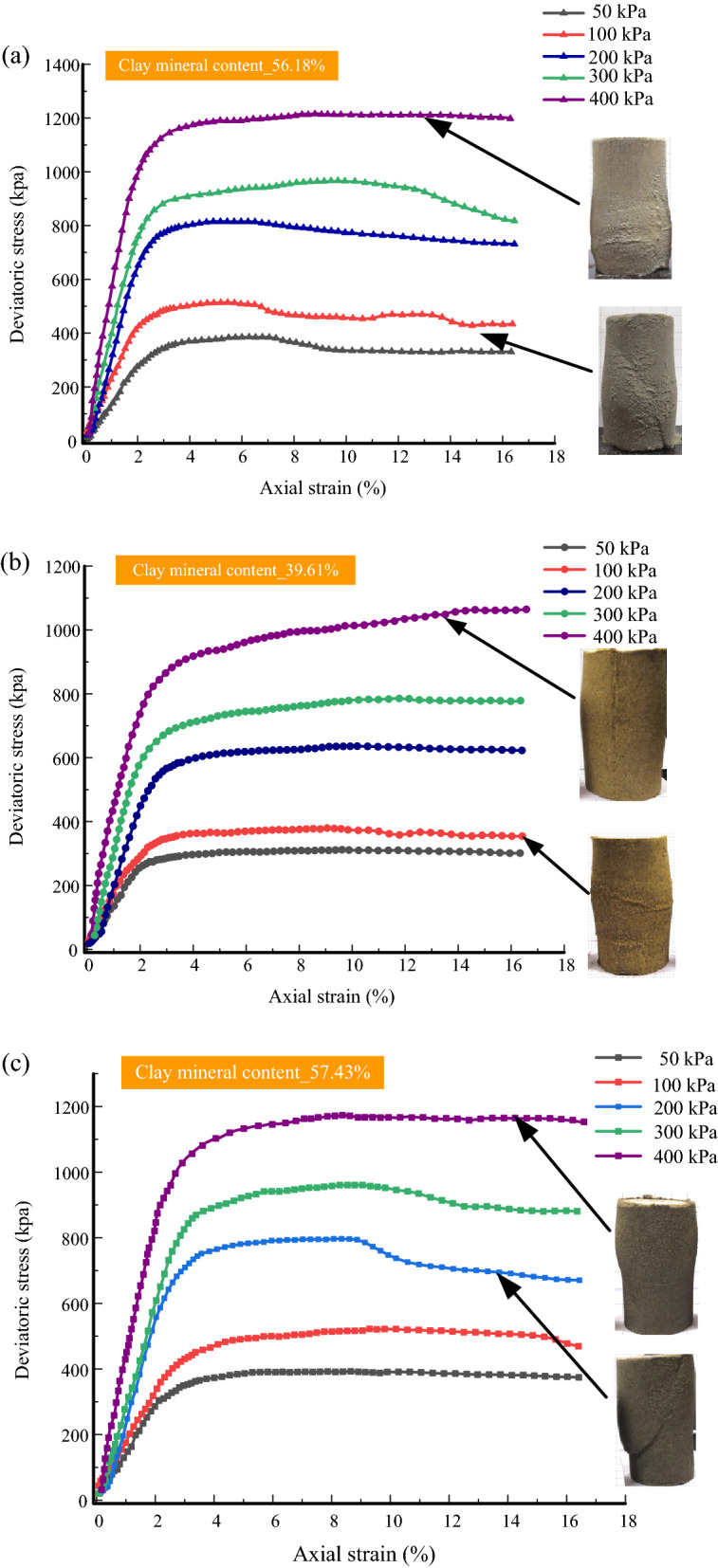
Stress–strain curves of tailings samples: (**a**) CTI, (**b**) CTII, (**c**) GT.

The stress–strain characteristics of geomaterials include nonlinearity, elastoplasticity and dilatancy. They are affected mainly by stress level, stress path, and stress history. Figure 8 presents the stress–strain curves and failure deformation of tailings samples. The features shown in stress–strain curves and deformation patterns include the following:

Initial elastic modulus and peak deviatoric stress increase. The axial strain corresponding to the rapid increase in deviatoric stress reduces with an increase of the confining pressure. The initial elastic modulus and shear strength are summarized in Table 4. For CTI samples under the confining pressure of 50 kPa, the stress–strain relationship curve is similar to that at 100 kPa. The deviatoric stress first increases with the axial strain. Then, it maintains a stable state after reaching the peak deviator stress. Then a stress drop occurs at the axial strain of 7%, which is strain softening. The homologous stress–strain relation also appeared in GT samples under confining pressure of 200 kPa and 300 kPa. A strain localization-shear band^22,23^ can be observed and presents a shear slip deformation. Under confining pressure of 200 kPa, the peak deviator stress is followed by a slow descent of the deviatoric stress. The deviatoric stress drops obviously at large strain under confining pressure of 300 kPa. The stress–strain relation of the sample is strain hardening, and there is no strain localization in the specimen. It shows a bulging deformation under confining pressure of 400 kPa. For CTII samples under confining pressures of 50 kPa and 100 kPa, the samples exhibit strain softening. Under the confining pressure of 200 kPa, deviatoric stress reaches a peak, and then it always maintains a plateau, called a quasi-steady state^24^. Under confining pressure of more than 200 kPa, strain hardening occurs, and the failure for the specimen is less evident with a bulging deformation. For GT samples, the strain-softening occurs under confining pressures of less than 400 kPa. It is in a quasi-steady state under confining pressure of 400 kPa.

**Table 4:** Initial elastic modulus and shear strength of tailings (σ = confining pressure).

Tailings types	Initial elastic modulus (MPa)					Shear strength (kPa)				
	σ = 50 kPa	σ = 100 kPa	σ = 200 kPa	σ = 300 kPa	σ = 400 kPa	σ = 50 kPa	σ = 100 kPa	σ = 200 kPa	σ = 300 kPa	σ = 400 kPa
CTI	11.2	15.1	26.7	33.8	42.5	385.4	513.8	814.7	964.8	1209.6
CTII	9.8	12.5	20.1	23.4	29.5	311.9	379.5	635.9	785.3	1064.5
GT	10.4	13.6	27.2	29.8	41.4	392.2	522.3	796.7	960.6	1173.3

Based on the mineral content of various tailings and the above analysis, the stress–strain relationship of the tailings is affected by the clay mineral content. The strain hardening is more likely to occur under high pressure in the undrained shearing test. As the content of clay minerals increases, the confining pressure required for strain hardening increases. With the increase of confining pressure, the stress–strain relationship gradually changes from strain-softening to strain-hardening, and there is a quasi-steady state between them. The more severe the strain softening, the more obvious the shear band. The tailings present a slight bulging failure under high confining pressure. The strain localization is more pronounced with an increase in the content of clay minerals. The bulging deformation decreases with increasing confining pressure, which implies that the tailings will shrink failure after being sheared at a higher confining pressure because of the limitation of rotation and slip of particles. This is consistent with previous research^25^.

### Influence of clay content on stress path

The track formed by the point representing the stress state in the stress-space is called the stress path^26^. For the consolidated undrained test, the variables used to depict the effective stress path in stress-space are the average effective principal stresses $p^{\prime}$ for the abscissa and the average deviator stress $q^{\prime}$ for the ordinate. They are defined as follows:

2 $$p^{\prime} = (\sigma_{1} ^{\prime} + \sigma_{2} ^{\prime} + \sigma_{3} ^{\prime})/3$$

where $$\sigma_{2} ^{\prime} = \sigma_{3} ^{\prime}$$.

3 $$q^{\prime} = (\sigma_{1} ^{\prime} - \sigma_{3} ^{\prime})/2$$

**Figure 9:**
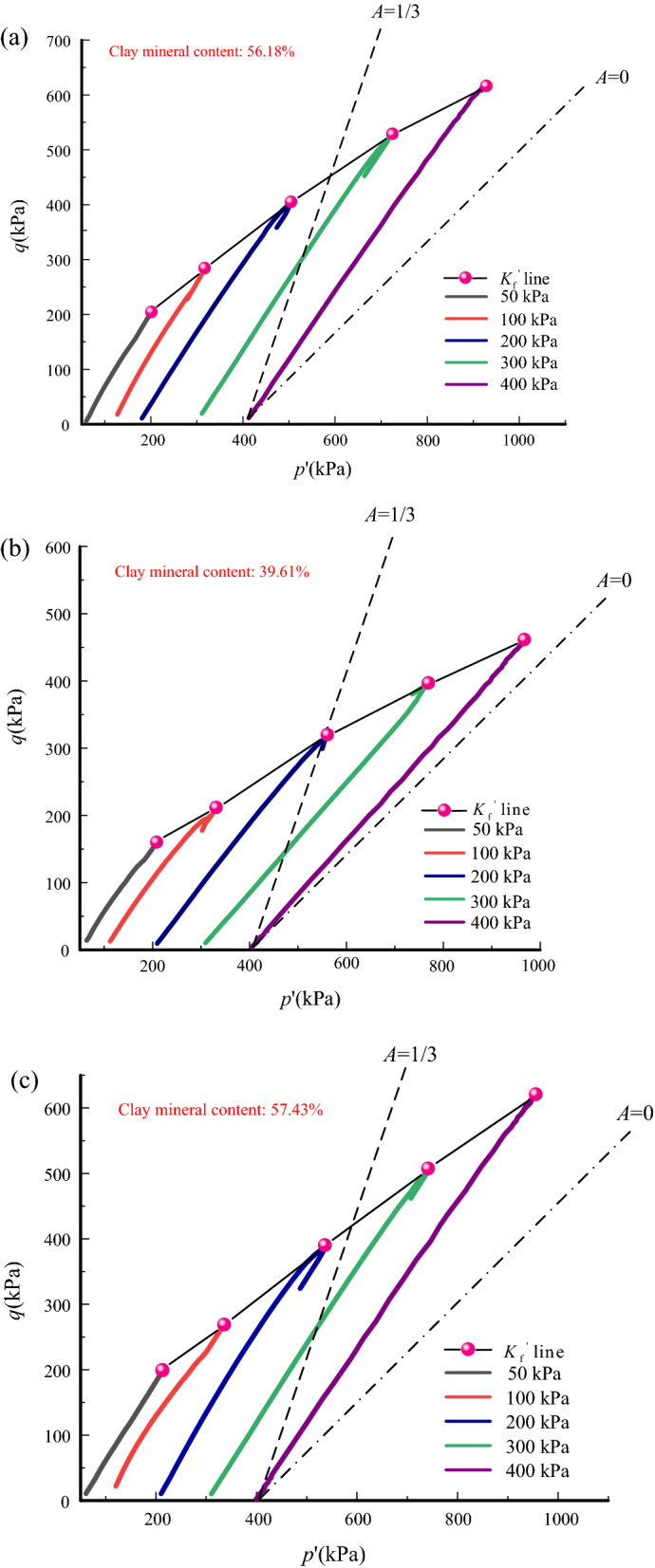
Stress path and *K*_f_ line of tailings samples: (**a**) CTI, (**b**) CTII, (**c**) GT.

Figure 9 shows the stress path of all tailings. These stress paths all are parallel to each other under different confining pressures. They present a linear growth, indicating that the pore pressure coefficient is unchanged during the shearing. A decline occurs in all the stress paths after the peak under confining pressures of 200 kPa and 300 kPa. It is mainly determined by strain softening. Only for CTII under the confining pressure of 300 kPa does the stress path fall to the left after the peak. The reason is that the pore pressure has a more prominent influence on the stress path than the deviatoric stress. Therefore, the pore pressure coefficient has a significant influence on the stress path. It can be observed from Fig. 9 that the pore pressure coefficients *A* of all tailings range from 0 to 1/3, indicating that dilatation occurs during the shearing. This explains that the deformation pattern of tailings samples shows bulging. The pore pressure coefficient of CTII is significantly lower than that of the other two tailings types, which may be due to its higher content of non-clay minerals, resulting in faster pore pressure dissipation.

The failure principal stress line^27^, that is, the *K*_f_ line, refers to the straight line below the strength envelope through the vertices of the Mohr circles, which can be obtained by connecting the average peak deviator stress point in stress-space under different confining pressures. The $K_{{\text{f}}}$ line of CTII is at the lowest position according to the value of the average peak deviator stress, and those of CTI and GT are relatively close. This is chiefly dominated by the same pore pressure coefficient and content of clay minerals. However, the clay mineral content of GT is slightly higher, its pore pressure coefficient is higher, and its overall $K_{{\text{f}}}$ line is the highest.

Therefore, the higher the clay mineral content, the larger the pore pressure coefficient. This is due to the higher viscosity between clay minerals and water and the agglomeration of clay minerals, leading to a less effective pore structure. This verifies the findings that the pore pressure dissipation is controlled by the effective pore structure^28–30^.

### Influence of clay content on shear strength

**Figure 10:**
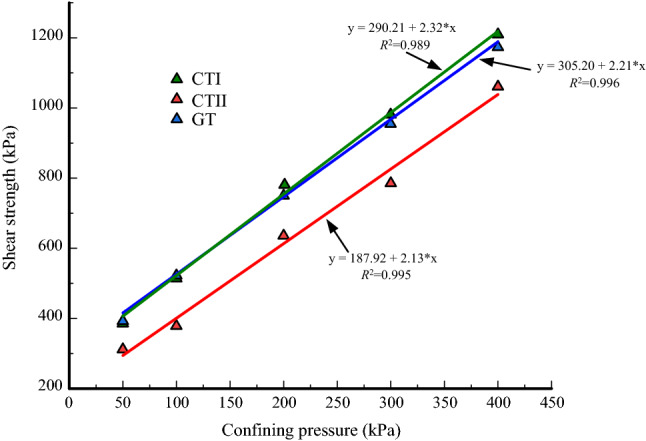
Shear strength of tailings samples.

For samples with strain softening, the peak deviator stress is the shear strength. For samples with strain hardening, the deviator stress corresponding to the axial strain of 15% represents the shear strength. Figure 10 shows the shear strength of tailings containing different minerals as a function of confining pressure. According to the Mohr–Coulomb strength criterion, the relation between the confining pressure and the shear strength in a linear relationship.

4 $$(\sigma_{1} - \sigma_{3} )_{f} = \frac{2}{1 - \sin \varphi }(c\cos \varphi + \sigma_{3} \sin \varphi )$$

where $(\sigma_{1} - \sigma_{3} )_{f}$ is shear strength, $c$ is the cohesion, $\varphi$ is the friction angle, $\sigma_{{1}}$ is the axial stress, $\sigma_{3}$ is the confining pressure.

The shear strengths of CTI and GT are nearly the same. They are higher than that of CTII. This suggests that the effect of clay mineral content on shear strength is similar to that on the failure principal stress line. The clay mineral content directly affects the strength parameters that determine the shear strength. The strength parameters are generally obtained from the strength envelope. There is always pore water pressure during undrained shear. Therefore, the total strength index and effective strength index can be calculated in the corresponding coordinate space. By Coulomb shear strength theory, the strength envelope can be expressed by Eqs. (5) and (6).

5 $$\tau_{{\text{f}}} = c_{{{\text{cu}}}} + \sigma \tan \varphi_{{{\text{cu}}}}$$

6 $$\tau_{{\text{f}}} = c^{\prime} + \sigma \tan \varphi ^{\prime}$$

where $\tau_{{\text{f}}}$ is shear strength, $c_{{{\text{cu}}}}$ is total cohesion, $\varphi_{{{\text{cu}}}}$ is total internal friction angle, $c^{\prime}$ is effective cohesion, $\varphi ^{\prime}$ is effective internal friction angle.

**Figure 11:**
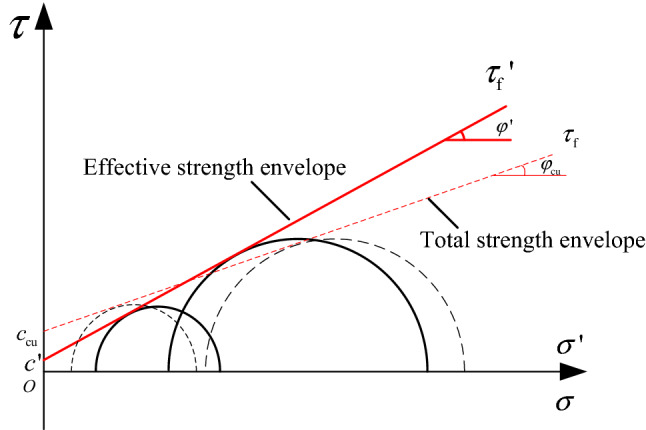
Schematic diagram of total strength and effective strength envelope. Red dashed line drawn in σ − τ space. Red solid line drawn in σ' − τ space.

**Figure 12:**
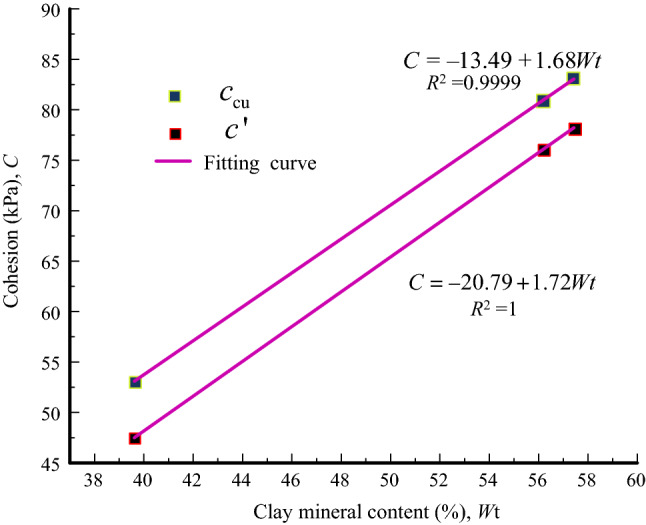
Relationship between cohesion and clay mineral content.

Figure 11 presents the schematic diagram of total strength and effective strength envelope. The total strength envelope is constructed through the tangent points of the total Mohr circles. The strength parameters of the tailings are summarized in Table 4. The cohesion of the three tailings is quite different. Based on this, the relationship between cohesion and clay mineral content can be established and is shown in Fig. 12. The clay mineral content and the cohesion have a linear relationship. The changes of total cohesion and effective cohesion with the increase of clay mineral content are similar. Their fitting straight lines are close to parallel, indicating that the initial pore water pressures of all tailings are approximately the same. However, this result is inconsistent with the previous conclusion that the cohesion first increases and then decreases with the increase of clay mineral content^31^. The reasons for this result are: (1) the clay mineral content of the tailings samples used in this study have not yet reached the optimal content. (2) The cohesive strength may not only be related to the content of clay minerals, but also the species of clay minerals.

From Table 5, although the internal friction angle first increases and then decreases with the content of clay minerals, the internal friction angles are restricted within a range between 31°and 33°, showing little change, regardless of the effective or total strength index. Generally speaking, the change of internal friction angle mainly depends on that of the clay mineral type, such as montmorillonite^32^ and kaolinite^33^. However, it has little correlation with the overall clay mineral content^34^.

**Table 5:** Shear strength parameters of tailings.

Tailings	$c_{{{\text{cu}}}}$ (kPa)	$\varphi_{{{\text{cu}}}}$ (°)	$c^{\prime}$ (kPa)	$\varphi ^{\prime}$ (°)
CTI	80.9	32.1	76.1	33
CTII	53.1	31	47.5	32
GT	83.1	31.5	78.2	32.8

### Microstructure analysis

Scanning electron microscope (SEM) is a standard method for studying the microstructure of geotechnical materials^35^. In accordance with the gradation of tailings samples, SEM scanning using 1000 times magnification is used to characterize the microscopic surface morphology of the tailings. The energy dispersive X-ray spectrometry (EDS) analysis is conducted at a certain position of the SEM images to determine the change in the tailings energy spectrum. The corresponding spatial positions are marked 1, 2 and 3, respectively.

**Figure 13:**
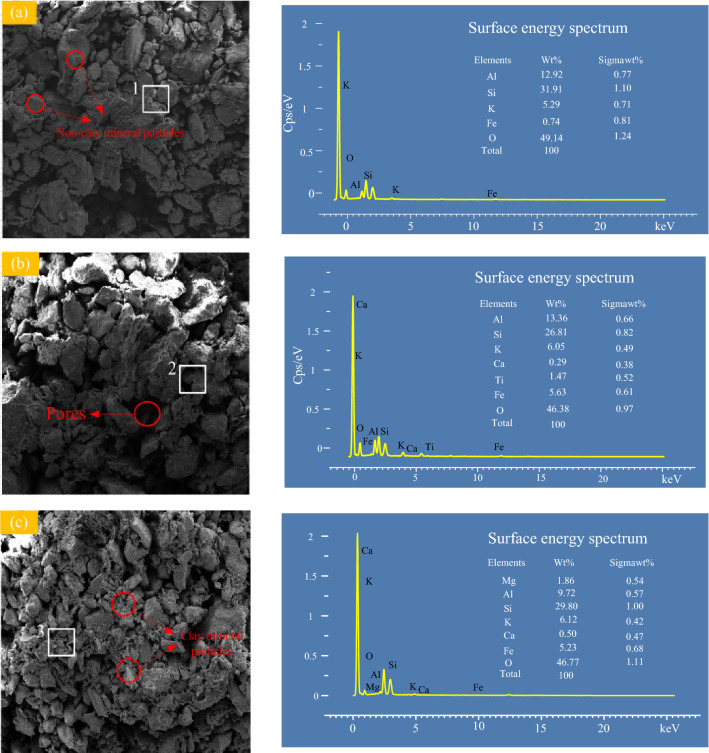
SEM images and energy spectrum of tailings. (**a**) CTI; (**b**) CTII; (**c**) GT.

Figure 13 illustrates the microstructure and energy spectrum of all tailings under a 1000 times magnification. The particles of CTI and GT samples have more obvious bonding, forming clusters, and there are needle-like structures on their surface. The reason is that there is a certain cohesion between the clay mineral particles, between the clay mineral and the non-clay mineral particles. The particles combine to shape inclusions in the tailings. The surface of CTII particles is smoother, and its particle fragments are mainly layered and flaky. The bond between particles is not strong. The pores between particles are large and relatively loose, and the distribution of clusters formed by clay minerals is more uniform. These results indicate that the higher the content of clay minerals, the larger the cohesion, which is consistent with the conclusions drawn from previous mechanical strength analyses^36^. The EDS analysis results show that the element distribution on the surface of the sample after the test is not much different from that before the test, indicating that the tailings sample has not reacted chemically after the addition of water. It confirms that the mechanical properties of tailings mainly are affected by the initial clay content based on the same particle gradation and similar clay components.

### Analysis of the effect of clay content on mechanical properties

Nitrogen adsorption is an effective method to study the microfabric characteristics of solid materials. Assuming that the nitrogen adsorbed on the surface of particles is a molecular layer, the specific surface area can be expressed by Eq. (7):

7 $$S_{{\text{g}}} = \frac{{N\delta V_{m} }}{22400w}$$

where *N* is Avogadro constant, and the number of gas molecules per unit mass is 6.024 × 10^23^; $\delta$ is the cross-sectional area of a nitrogen molecule. $V_{m}$ is a single layer adsorption volume of nitrogen on the inner surface of sample pores. $w$ is the quantity of the sample tested.

However, in most cases, nitrogen is not absorbed in a single layer in the material pores. Assuming that the adsorption heat of the first layer is a constant, and the adsorption heat of other layers is a different value, then based on thermodynamic and kinetic analysis, the real volume of nitrogen in the material pores can be calculated by using the BET (Brunauer, Emmett, and Teller) Equation^37,38^:

8 $$\frac{P}{{V(P_{0} - P)}} = \frac{1}{{V_{m} C}} + \frac{C - 1}{{V_{m} C}}\left( {\frac{P}{{P_{0} }}} \right)$$

where $V$ is the real volume of nitrogen adsorbed in pores of a unit mass sample; $P$ is nitrogen partial pressure; $P_{0}$ is the saturated vapour pressure of nitrogen at liquid nitrogen temperature; $C$ is adsorption heat constant, the larger its value, the stronger the adsorption capacity; the range of $P/P_{0}$ is 0.05–0.35.

**Figure 14:**
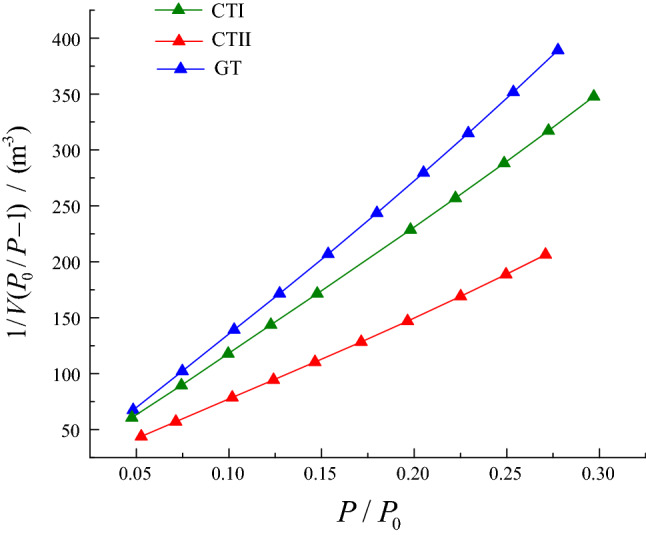
The BET curves of tailings.

Figure 14 shows the BET curves of all tailings. According to Eq. (8), the monolayer adsorbate volume $V_{m}$ and the adsorption heat constant $C$ can be obtained by the slope and intercept of the curves. Combined with Eq. (7), the specific surface area $S_{{\text{g}}}$ of the material can be obtained. The calculated specific surface areas of CTI, CTII and GT are 3.03, 4.69, and 2.51m^2^/g, respectively. Specific surface area is negatively related to the content of clay minerals in the case of the same particle size distribution. Since the more clay particles, the larger aggregates directly results in higher cohesion of tailings. The calculated adsorption heat constants of CTI, CTII and GT are 330.94, 223.91 and 336.99, respectively. This shows that clay mineral particles have a stronger adsorption capacity, associated with the electronic shell on the surface of mineral particles.

Nitrogen has isothermal adsorption characteristics^39^. Assuming that the capillary pores on the surface of the material are cylindrical, the pore diameter corresponding to the pressure at which nitrogen is desorbed from the condensed state, can be obtained by the Kelvin Equation^40^:

9 $$r_{k} = \frac{{ - 2\gamma {\text{V}}_{m} }}{{RT\ln (P/P_{0} )}}$$

where $r_{k}$ is the pore diameter, $\gamma$ is the surface tension of nitrogen at boiling point, ${\text{V}}_{m}$ is Molar volume of liquid nitrogen, $R$ is gas constant, $T$ is the boiling point of nitrogen.

BJH (Barret-Joyner-Halenda) method^41^ is commonly used for nitrogen adsorption to measure pore size distribution. During nitrogen desorption, the equation for the change of pore volume in the range of $P/P_{0}$ from 1 to 0, is as follows:

10 $$\Delta V_{pi} { = }\left( {\frac{{\overline{{r_{pi} }} }}{{\overline{{r_{ki} }} }}} \right)^{2} \left( {\Delta V_{ki} - 2\Delta t_{i} \mathop \Sigma \limits_{j = 1}^{i - 1} \frac{{\Delta V_{pj} }}{{\overline{{r_{pj} }} }}} \right)$$

where $V_{pi}$ is the measured adsorption pore volume corresponding to the pore diameter $r_{pi}$, $\Delta V_{ki}$ is the volume of liquid nitrogen converted from the amount of nitrogen desorbed from the solid surface when the relative pressure drops from $P_{i - 1}$ to $P_{i}$. $\left( {\frac{{\overline{{r_{pi} }} }}{{\overline{{r_{ki} }} }}} \right)^{2}$ is a conversion fraction.

The nitrogen adsorption test results are usually expressed in terms of the calculated cumulative pore volume and the differential of pore volume to the logarithm of pore size for all calculated pore diameters^42^:

11 $$V_{cumulative} = \sum {\Delta V_{pi} }$$

12 $$V_{\log - differential} = \frac{{dV_{pi} }}{{d(\log r_{pi} )}}$$

**Figure 15:**
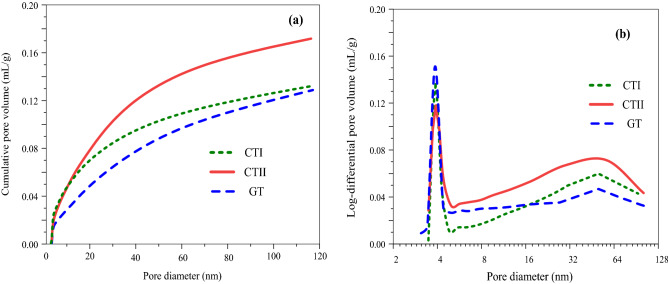
Effect of mineral content on nitrogen adsorption test results of tailings.

Figure 15 shows the pore size distribution of tailings samples by nitrogen adsorption test. It can be seen from Fig. 15a that as the pore size increases, the cumulative pore volume increases rapidly and then tends to flatten, indicating that almost all pore sizes are within the detectable range. For samples with the same initial density, the cumulative pore volume should eventually approach the same value. However, the ultimate cumulative pore volume of tailings decreases with the increase of clay mineral content. This result may be due to the fact that due to the higher content of clay minerals, capillary water pressure increases during the consolidation phase, providing additional impetus for pore shrinkage.

Figure 15b presents the log-differential pore volume of tailings. All samples have a bimodal pore size distribution, with two major pore sizes. They can be divided into macroscopic pores (between aggregation and aggregation) and microscopic pores (aggregated interior). The duplicate porosity feature usually occurs when the soil samples are compressed at the optimal moisture content^43,44^. Furthermore, the two main pore sizes of all tailing samples are similar, indicating that the bimodal pore size distribution mainly depends on the initial gradation of the material. The rise of the proportion of dominant micropores and the reduction of that of dominant macropores is attributed to the better coating of tailings because of higher clay mineral content.

From Fig. 15, the shear strength of tailings is greatly related to the clay content. For instance, as the content of clay minerals increases, the proportion of micropores increases. It is generally manifested as the reduction of porosity and the elaboration of pore size. They both lead to an increase in the shear strength, as evidenced in this study. However, what calls for special attention is that the effect of clay content on the shear strength is very complicated. Further microstructural analysis and chemical research on these materials are required. More deeply, the shear strength of soils is determined by many aspects, not only correlated with their fabric, but also with the cementing of the particles, the crushing strength of the particles, and the rearrangement of the particles.

## Conclusions

According to the test results presented in this paper, several conclusions can be drawn:

- Under lower confining pressure, strain-softening is more likely to occur when tailings are subjected to undrained shear. A shear band failure is observed for these samples. Under higher confining pressure, strain hardening is more likely. A bulging deformation failure is observed for these samples. The confining pressure for strain hardening increases. The strain localization in samples is more significant with increasing clay mineral content.
- Based on the same particle gradation, the shear strength of tailings increases with the increase of clay mineral content due to more cohesion and almost constant internal friction angle. The cohesion is linearly related to clay mineral content. The impacts on the effective strength index and the total strength index are similar.
- Scanning electron microscope was used to analyze the influence of clay mineral content on the mechanical properties of tailings from a microscopic point of view. The increase in the content of clay minerals promotes the bonding between the tailings particles, and the surface of the particles presents a needle-like structure. The non-clay mineral particles are mainly layered and flaky. The bond between particles is not strong, and the apparent porosity is also large.
- The study of the microfabric of tailings through pore size distribution helps explain the influence of clay mineral content on the mechanical properties of tailings. A dual pore size distribution feature was found in the compacted tailings samples. The main two pore sizes are dominant by initial gradation. The proportion of micropores and macropores changes with the clay mineral content, which can help in understanding the soil materials behaviour from a micro perspective. It confirms the accuracy of the test results. Further research on the influence of clay mineral content on the mechanical behaviour of tailings from multiple aspects should be performed, which will be beneficial for the stability and management of tailings dams around the world.

## Data Availability

Some or all data, models, or code that support the findings of this study are available from the corresponding author upon reasonable request. All data, models, and code generated or used during the study appear in the submitted article.
